# Impact of Plasmids, Including Those EncodingVirB4/D4 Type IV Secretion Systems, on *Salmonella enterica* serovar Heidelberg Virulence in Macrophages and Epithelial Cells

**DOI:** 10.1371/journal.pone.0077866

**Published:** 2013-10-03

**Authors:** Kuppan Gokulan, Sangeeta Khare, Anthony W. Rooney, Jing Han, Aaron M. Lynne, Steven L. Foley

**Affiliations:** 1 Division of Microbiology, FDA National Center for Toxicological Research, Jefferson, Arkansas, United States of America; 2 Department of Chemistry, University of Minnesota-Morris, Morris, Minnesota, United States of America; 3 Department of Biological Sciences, Sam Houston State University, Huntsville, Texas, United States of America; Wadsworth Center, New York State Dept. Health, United States of America

## Abstract

*Salmonella enterica* serovar Heidelberg (*S.* Heidelberg) can cause foodborne illness in humans following the consumption of contaminated meat and poultry products. Recent studies from our laboratory have demonstrated that certain *S.* Heidelberg isolated from food-animal sources harbor multiple transmissible plasmids with genes that encode antimicrobial resistance, virulence and a VirB4/D4 type-IV secretion system. This study examines the potential role of these transmissible plasmids in bacterial uptake and survival in intestinal epithelial cells and macrophages, and the molecular basis of host immune system modulation that may be associated with disease progression. A series of transconjugant and transformant strains were developed with different combinations of the plasmids to determine the roles of the individual and combinations of plasmids on virulence. Overall the *Salmonella* strains containing the VirB/D4 T4SS plasmids entered and survived in epithelial cells and macrophages to a greater degree than those without the plasmid, even though they carried other plasmid types. During entry in macrophages, the VirB/D4 T4SS encoding genes are up-regulated in a time-dependent fashion. When the potential mechanisms for increased virulence were examined using an antibacterial Response PCR Array, the strain containing the T4SS down regulated several host innate immune response genes which likely contributed to the increased uptake and survival within macrophages and epithelial cells.

## Introduction

Pathogenic bacteria account for an estimated 3.6 million foodborne illnesses each year in the United States, with nearly a third (1.04 million) due to nontyphoidal *Salmonella enterica* [1]. *Salmonella* infections alone contribute around 35% of hospitalizations and 64% of deaths associated with bacterial foodborne pathogens [1]. *Salmonella* can contaminate a wide range of food products such as meat and poultry products, eggs, fruits, vegetables and nut-based foods [2]. Of the *Salmonella*, serovar Heidelberg generally ranks among the top 5 most commonly identified serovars associated with human salmonellosis in the U.S. [3].

In 2011 alone, *S. enterica* serovar Heidelberg (*S.* Heidelberg) caused several outbreaks and sporadic cases of gastroenteritis in the U.S. and Canada [4,5]. For example, a multistate *S.* Heidelberg outbreak that was traced back to contaminated ground turkey caused at least 130 infections in 34 states [4]. Of added concern, the strain associated with this outbreak was resistant to multiple antimicrobial agents, including ampicillin, tetracycline and in some cases streptomycin and gentamicin [4]. Several other researchers have also detected multidrug resistant *S.* Heidelberg, including resistance to clinically important third generation cephalosporins, in foods, food production and processing environments, and isolates from human infections [6-9], which could limit the treatment options for patients with severe infections. *S.* Heidelberg is among the most detected serovars isolated from turkey-, chicken- and swine-related sources according to data from USDA’s National Veterinary Services Laboratory [3]. In addition, *S.* Heidelberg has been one of the most common serotypes isolated from retail meats as part of the National Antimicrobial Resistance Monitoring System (NARMS) [10].

*S.* Heidelberg are responsible for 3.5% of all reported *Salmonella* infections in the U.S., yet leads to approximately 7% of the *Salmonella*-related deaths; the second highest percentage after *S. enterica* serovar Typhimurium [3,11]. *S.* Heidelberg can cause severe extra-intestinal infections including septicemia and those of the organ systems [12,13]. In these severe infections, antimicrobial resistance is a major public health concern, because antimicrobial therapy is generally warranted [14]. Many of the genes associated with antimicrobial resistance in *Salmonella* are located on plasmids and in several instances genes that contribute to virulence are also plasmid encoded [15-17].

Much of the work elucidating *Salmonella* pathogenicity has been focused on serovar Typhimurium. Infections occur when the organisms are able to survive the low pH of the upper gastrointestinal tract and attach and invade intestinal epithelial cells. The invasion of the cells is reliant upon the expression of a type III secretion system (T3SS) encoded by Salmonella pathogenicity island (SPI) 1 [18]. Once internalized in Salmonella containing vacuoles (SCVs) a second T3SS encoded by SPI 2 is expressed that allows for *Salmonella* survival in the host cells [19]. Invasive strains can also enter immune cells, such as macrophages or dendritic cells, using the same T3SS-associated mechanisms and can lead to more severe disease manifestations such as septicemia and organ systems infections [20]. While much is known about *S. Typhimurium* virulence, there are gaps in the understanding of how different serovars lead to infection and whether putative virulence factors located on plasmids impact the ability of *Salmonella* to infect different hosts.

To begin to close some of the knowledge gaps, plasmids from five multidrug resistant *S.* Heidelberg strains were sequenced [21]. Each strain had plasmids associated with the observed antimicrobial resistance phenotypes and three of the strains had additional 32-35 kb plasmids with genes that appeared to be associated with a VirB4/D4 type IV secretion system (T4SS). These VirB/D4 T4SS plasmids have not been previously described in *Salmonella*, however there are similar plasmid sequences deposited in GenBank from *Shigella boydii, Citrobacter rodentium* and *Escherichia coli* (accession numbers: CP001059.1, FN543504 and JN194214.1, respectively). Consequently, the function of these T4SS in *Salmonella* has not been previously described. In other intracellular pathogens, analogous T4SS play a significant role in pathogenesis [22-24] including intracellular replication [25], targeting to endoplasmic reticulum (ER) [26], and maintaining infection in animals [27]. In addition to the T4SS containing plasmids, each of the three *S.* Heidelberg isolates also carried incompatibility group (Inc) A/C resistance plasmids and two carried an IncFIB plasmids. IncFIB plasmids have been associated with virulence in avian pathogenic *E. coli* and S. Kentucky isolated from poultry [28]. Therefore to gain a better understanding of the impact of the T4SS and co-located plasmids on *Salmonella* virulence, the present study was conducted to evaluate the VirB4/D4 T4SS containing plasmids and address the potential involvement of plasmid encoded factors on bacterial uptake, colonization, survival and host gene expression profile during early invasion.

## Materials and Methods

### Bacterial strains

Three *S.* Heidelberg isolates containing multiple plasmids were selected based on the results of plasmid sequencing experiments [21]. These isolates were numbered 146 (contains VirB/D4 T4SS, IncA/C, an untypeable resistance and IncI1 plasmids), 163 and 696 (both contain VirB/D4 T4SS, IncA/C and IncFIB plasmids). *S.* Heidelberg isolate 819 [8] and *E. coli* One Shot TOP10 (Invitrogen, Carlsbad, CA) were used as the recipient strains for conjugation and transformation studies, respectively. To generate transconjugants with different plasmid compositions, the plasmid donor strains (146, 163 and 696) were filter mated with 819 as described previously [8] and the transconjugants selected by plating on nutrient agar with 32 µg/ml of ampicillin and 64 µg/ml of nalidixic acid. In addition, plasmids were isolated from strain 163 using the methods described by Wang and Rossman [29] and the Wizard Plus SV Minipreps kit (Promega, Madison, WI). The isolated plasmid DNA was transformed into *E. coli* One Shot TOP10 cells as described by the manufacturer and positive transformants were selected and screened for the presence of each of the plasmids using PCR primers and conditions previously described for *bla*_CMY_ (IncA/C marker), s*itA* (IncFIB marker) and *virB4* (VirB/D4 marker) [30,31]. Plasmids were also isolated from the transconjugant and transformant strains [29] and separated as described previously [8] to further confirm the presence of the plasmids in the strains evaluated. Table 1 describes the strain constructs used in the study. The bacterial strains were maintained at -80°C in brain heart infusion broth with 20% glycerol. For the experiments, bacterial strains were streaked onto nutrient agar and grown overnight at 37°C. A single colony was used to inoculate 2 ml Luria Broth (LB) media at 37°C with shaking at 250 RPM for overnight. The following day, 50 µl of overnight culture was inoculated into 3 ml fresh LB media with shaking at 250 RPM at 37°C until the culture reached mid log phase (A_600_ between 0.6 to 0.8).

**Table 1:** Bacterial strain identities and plasmid content of strains used in this study.

Bacterial strains	IncA/C Plasmid	IncFIB Plasmid	VirD4/B4 Plasmid
*S.* Heidelberg Wildtype			
146	**+**	**-**	**+**
163	**+**	**+**	**+**
696	**+**	**+**	**+**
819	**-**	**-**	**-**
*S.* Heidelberg Transconjugants			
TC-A	**+**	**-**	**-**
TC-AF	**+**	**+**	**-**
TC-AFV	**+**	**+**	**+**
*E. coli* Recipient			
*E. coli* Top10	**-**	**-**	**-**
*E. coli* Transformants			
TF-V	**-**	**-**	**+**
TF-AFV	**+**	**+**	**+**

### Macrophage culture

J774 mouse macrophage cells (TIB-67; American Type Culture Collection (ATCC), Manassas, VA) were grown in 75mm culture flasks in Dulbecco’s modified Eagle’s medium (DMEM) supplemented with 2mM glutamine, 10% fetal bovine serum (FBS), penicillin (100 units/ml) and streptomycin (100 µg/ml). The cells were cultured in 37°C with 5% CO_2_ in 95% humidity. Cells were maintained in the flask until they reached a complete monolayer. Upon confluence, cells were detached from the culture flask using cell scraper and washed with DMEM. The cell pellet was re-suspended in enriched DMEM and plated into 24-well culture plates (4 x 10^5^ cells per well). The culture plates were incubated at 37°C with 5% CO_2_ and 95% humidity for 48 hrs.

### Intestinal epithelial cells

Rat epithelial cells (CRL-1592; ATCC) were grown in DMEM containing 2.5 mM L-glutamine supplemented with 5% FBS and bovine insulin, in an atmosphere of 5% CO_2_, at 37°C. The cells were maintained in the culture flask until reaching a complete monolayer. Upon confluence, cells were detached from the flask by incubating with 0.25% Trypsin-EDTA solution at 37°C for 15 min. The detached cells were washed with DMEM and re-suspended in complete DMEM media. The epithelial cells were plated in 24-well culture plate with 5 x 10^4^ cells per well and the plates further incubated at 37°C with 5% CO_2_ and 95% humidity for 48 hrs.

### Bacterial uptake/invasion assays

Bacterial uptake/invasion assays were performed with a minor modification from previously a published protocol [32]. Mouse macrophage and rat intestinal epithelial cells were infected with each of the strains listed in Table 1 with an MOI (multiplicity of infection) of 1:200 (host cell: bacteria). Bacterial cells suspended in DMEM (8 x 10^7^ and 1 x 10^7^ cells/well) were added to the wells containing macrophages and epithelial cells, respectively. The plates were centrifuged for 500 RPM for 5 min and allowed to incubate for 1 hr at 37°C in 5% CO_2_. After 1 hr incubation the cells were washed with pre-warmed PBS (pH 7.4) three times to remove extracellular bacteria. The cells were incubated with 50 µg/ml of gentamicin (Life Technology, Grand Island, NY) for 1 hr at 37°C to kill the extracellular bacteria. After 1 hr, cells were washed again with PBS and incubated with 1% Triton X-100 for 5 min at 37°C for the lysis of eukaryotic cells. Aliquots were collected, serially diluted in PBS buffer, and plated onto LB agar plates and incubated at 37°C for 18 hrs, after which, the colony forming units (CFUs) were counted. For each experimental replicate, the assays were performed in duplicate and the experiment was repeated a total of five times. The mean and standard deviation were then calculated for the experiments. Macrophages and epithelial cells that were not infected with bacteria served as controls.

### Bacterial survival

For the bacterial survival assays, cells were infected with each of the bacteria strains (Table 1) for 1 hr as described for the bacterial uptake assay. After 1 hr, the cells were washed with pre-warmed PBS and incubated with 50 µg/ml of gentamicin for 24 hrs at 37°C. After 24 hrs, cells were washed with PBS and lysed with 1% Triton X-100. The number of bacteria surviving was determined by plating serial dilutions of the cell lysates as described above. The CFUs were counted after 18 hrs of incubation at 37°C. Each set of experiments were repeated a total of five times and the mean and standard deviations were calculated for the experiments.

### Gene expression of antibacterial response pathways

Macrophages were infected with *S.* Heidelberg isolates 819 and TC-AFV for 1 hr as described in the previous section. After 1 hr, cells were washed with PBS and 1ml of ice cold Tri-reagent (Molecular Research Center, Cincinnati, OH) was thoroughly mixed with the macrophages to extract the RNA from the macrophages as described by the manufacturer. The RNA was treated DNase (Ambion, Austin, TX), quantified using a ND-1000 spectrophotometer (NanoDrop Technologies, Wilmington, DE) and stored at -80°C until used. cDNA was generated by adding 2 µg of total RNA to a 100-µl reaction containing 2.5 mM random hexamer and reagents from a Reverse Transcription Kit (Applied Biosystems, Foster City, CA) as described by the manufacturer. Gene expression was performed using Antibacterial Response PCR Arrays (SA Biosciences/ Qiagen, Valencia, CA) in an Applied Biosystems 7500 DNA Sequence Detection System. Analysis of the PCR data was performed using PCR Array Data Analysis Software (SA Biosciences). Each of the analyses were repeated a total of three times and the mean and standard deviations were calculated for the experiments.

### Gene expression of T4SS genes during bacterial infection

Macrophages were grown in 75mm culture flasks (8 x 10^6^ cells per flask) as described above and infected with either *S.* Heidelberg isolate 819 or 696 (1.6 x 10^9^ bacteria) per flask for either 5 min, 30 min or 60 min. Each set of experiments was done in triplicate. After incubation, the macrophages were washed 3 times with PBS to remove extracellular bacteria. One ml of ice cold Tri-reagent was thoroughly mixed with the macrophages for cell lyses and RNA extraction from the macrophages and internalized bacteria as described by the manufacturer (Molecular Research Center). Total bacterial RNA from 1.6 x 10^9^ log phase bacteria grown in LB media (as described above) and host total RNA was extracted from 8 x 10^6^ macrophage cells using the Tri-reagent. RNA was treated DNase and quantified as described above and stored at -80°C until used. cDNA was generated according to the manufacturer’s protocols with the Transcriptor First Strand cDNA Kit (Roche Applied Science, Indianapolis, IN). Gene expression profiling of the T4SS encoding genes was performed using a panel of primers and corresponding universal probes (Universal Probe Library, Roche Applied Science) designed using the Assay Design Center software (Roche Applied Science; Table 2). Quantitative PCR reactions were done using the Applied Biosystems 7500 DNA Sequence Detection System and analyses performed using PCR Array Data Analysis Software (SA Biosciences). Each of the analyses were repeated a total of three times and the means and standard deviations were calculated for the experiments.

**Table 2:** Primers and probes used for the T4SS gene expression experiments.

**Genes**	**Forward primer**	**Reverse Primer**	Probe*
*virB1*	tcgagagtggcttcaatcct	tagctaccgcgttcccttt	5
*virB2*	ccagcttttgcagatggatt	accaagccctgttttgacc	120
*virB3*	cgggggtttatatcagcaag	tgagaataaaaactgcgacagg	20
*virB4*	cagagtaactgattctccggtgt	tctgtccggtatcatgattaaaaa	22
*virB5*	gattttaccagtaatcccgaagg	aggctccatacttgctgaaca	71
*virB6*	gggcgctttattggttctta	ttcaacacctgcacctgcta	56
*virB8*	aagaaaaatgcatggcgtgt	agccccaagacataaggaca	59
*virB9*	tttttaggttttaactcagggaaagt	ttcctgaccatctcttacagca	33
*virB10*	tgctcagtatggttcaggatgt	cgatctttaccaggtgcattatc	70
*virB11*	ggcgagaccaagccattac	catgcaggaggaaaaacgac	47
*virD2*	cgtggagaaaacatcgatca	tccctgcttactccagctt	77
*virD4*	cagagttttagcctttgttgcac	ccgcaacagggatgataaga	15
16SRNA	ttggtgaggtaacggctca	gctggtcatcctctcagacc	141

* Probe number from the Universal ProbeLibrary (Roche-Applied Sciences, Indianapolis, IN).

### Cell cytotoxicity assay

Macrophage or intestinal epithelial cells were infected as described above. After 1 hr, culture supernatant was collected and stored at -20°C until use. Cytotoxicity assays were performed using CytoTox 96 Non-Radioactive Cytotoxicity Assay kit (Promega). The cytotoxicity results were expressed in fold difference in lactate dehydrogenase (LDH) activity using the following formula: fold difference = cytotoxicity induced by bacterial uptake/ cytotoxicity of uninfected control cells. Each of the analyses were repeated a total of five times and the means and standard deviations were calculated for the experiments.

### Statistical methods

Statistical analyses for bacterial uptake, survival and cytotoxicity assays were done using GraphPad (GraphPad Software, Inc, La Jolla, CA). The unpaired t-test was used to calculate the statistical significance. For the pathway array experiments, statistical significance was calculated by using SA Biosciences online software. Differences were considered statistically significant at p<0.05.

## Results

The present study examined the role of plasmids in the bacterial uptake and survival of *S.* Heidelberg that were isolated from food animal sources. Each of the wild-type strains examined contained antimicrobial resistance plasmids and a plasmid encoding a VirB/D4 T4SS system. A series of transconjugants and transformant strains were developed with various plasmid constructs to evaluate the potential impact on virulence (Table 1). Transconjugants were generated with all three larger plasmids (IncA/C, IncFIB and VirB/D4), with IncA/C and IncFIB, and IncA/C and are named TC-AFV, TC-AF and TC-A, respectively. The Promega Wizard plasmid miniprep isolated the 34 kb VirB/D4 plasmid, but not the IncA/C and IncFIB plasmids, which was successfully transformed into the *E. coli* OneShot TOP10 cells without the IncA/C and IncFIB plasmids; this strain was named TF-V. Alternatively, all three larger plasmids from isolate 163 were transformed into the *E. coli* strain to form the strain (TF-AFV). PCR screening and plasmid isolation and separation verified the presence of the plasmids in the transconjugants and transformants. The details of these strains are provided in Table 1.

**Figure 1:**
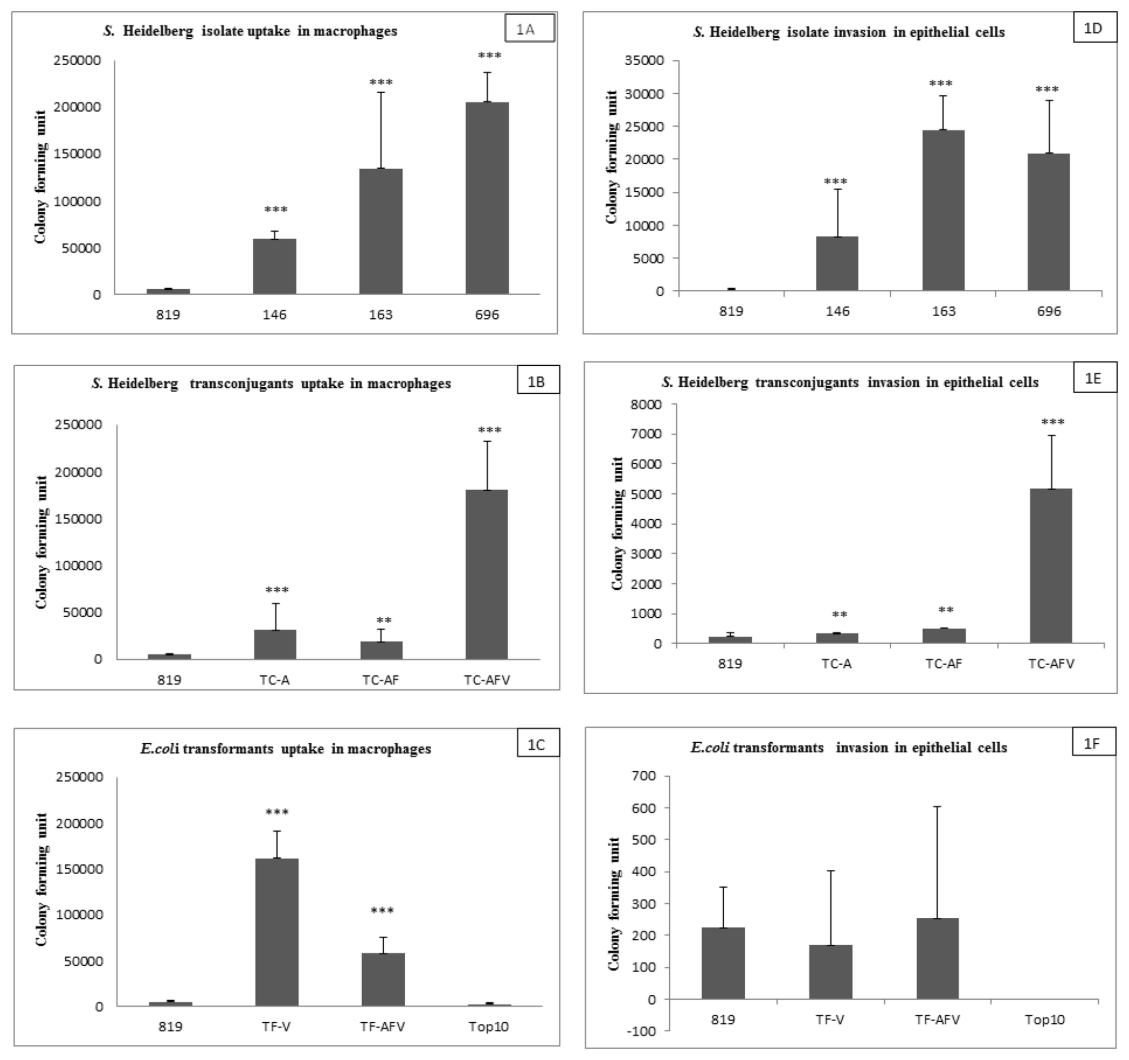
*In vitro* infection of mouse macrophages and epithelial cells with *S.* Heidelberg strains (146, 163, 696), transconjugates and transformants that harbor transmissible plasmids, T4SS genes and strains that lacks both (819 and *E. coli* One Shot TOP10). Bacterial uptake assay was performed 1 hr post infection. Panels A-C shows the number of CFUs of wildtype *S.* Heidelberg isolates (146, 163, 696 and 819), transconjugants and transformants 1 hr post infection of macrophages. Panels D-F shows the number of CFUs following a 1 hr infection of epithelial cells. Each data point represents the means and standard deviation for 5 individual experiments.

During the bacterial uptake process by macrophage, the wildtype isolates (146, 163 and 696) that contained the IncA/C and VirB/D4 plasmids showed significantly higher uptake (as determined by the number of CFUs counted after host cell lysis) than isolate 819, which lacked the plasmids (all with p<0.001; Figure 1A). This low level of bacterial uptake in 819 served as a baseline to evaluate the impact of plasmids on invasiveness and survival. While comparing the magnitude of bacterial uptake among *S.* Heidelberg isolates that harbor transmissible plasmids, isolates 696 and 163 showed higher levels of internalization than isolate 146 (p<0.05 and p<0.001, respectively) (Figure 1A). Isolate 146 lacks the IncFIB plasmid that was present in isolates 163 and 696. The baseline level bacterial uptake/invasion and survival by isolate 819 in epithelial cells and macrophages could be attributed towards with other virulence factors, such at those associated with SPI 1 and 2. Realtime-PCR analysis of the expression of SPI 1 and 2 genes (*sifA, pagC, invA* and *orgA*) in *S.* Heidelberg strains 146, 163, 696 and 819 showed that these genes were expressed at comparable level in all the strains (data not shown).

When the transconjugants and transformants were evaluated in the macrophage system, TC-AFV, containing all three plasmids was found to be highly internalized as compared to the recipient isolate 819 (p<0.001). The level of bacterial uptake was very similar to isolate 696, the plasmid donor for the strain. The uptake and survival of the two other transconjugants, TC-AF and TC-A, was significantly lower than for TC-AFV (Figure 1B). The results of uptake by *E. coli* transformants show that strain TF-V was significantly more able to enter macrophages (p<0.001) than 819 and the *E. coli* recipient strain that lacked the VirB4/D4 plasmid (Figure 1C). Interestingly, strain TF-AFV that received all three plasmids produced a lower number of CFUs following uptake of macrophages, indicating a lower level of potential invasiveness than TF-V. Even so, strain TF-AFV had a significantly higher level of internalization than the 819 and its *E. coli* recipient strain (Figure 1C).

In the evaluation of the bacterial invasion of intestinal epithelial cells all three *S.* Heidelberg isolates that carry mobile plasmids were more highly invasive (isolate 146= p<0.05, isolate 163= p<0.001 and isolate 696= p<0.002) than isolate 819 (Figure 1D). Similar to the macrophage results, TC-AFV was significantly more able to enter and survive in epithelial cells than isolate 819 and the other transconjugants formed (Figure 1E). The *E. coli* transformants (TF-AFV and TF-V) were not significantly more able to invade the epithelial cells than 819 (Figure 1F). The pattern of invasiveness for the *Salmonella* isolates in epithelial cells was similar to the results for the macrophages, however, the number of bacteria surviving (CFUs) was generally greater in the macrophage experiments, which may correlate to the higher number of cells (macrophages and bacteria) used in the experiments (Figure 1A-F).

**Figure 2:**
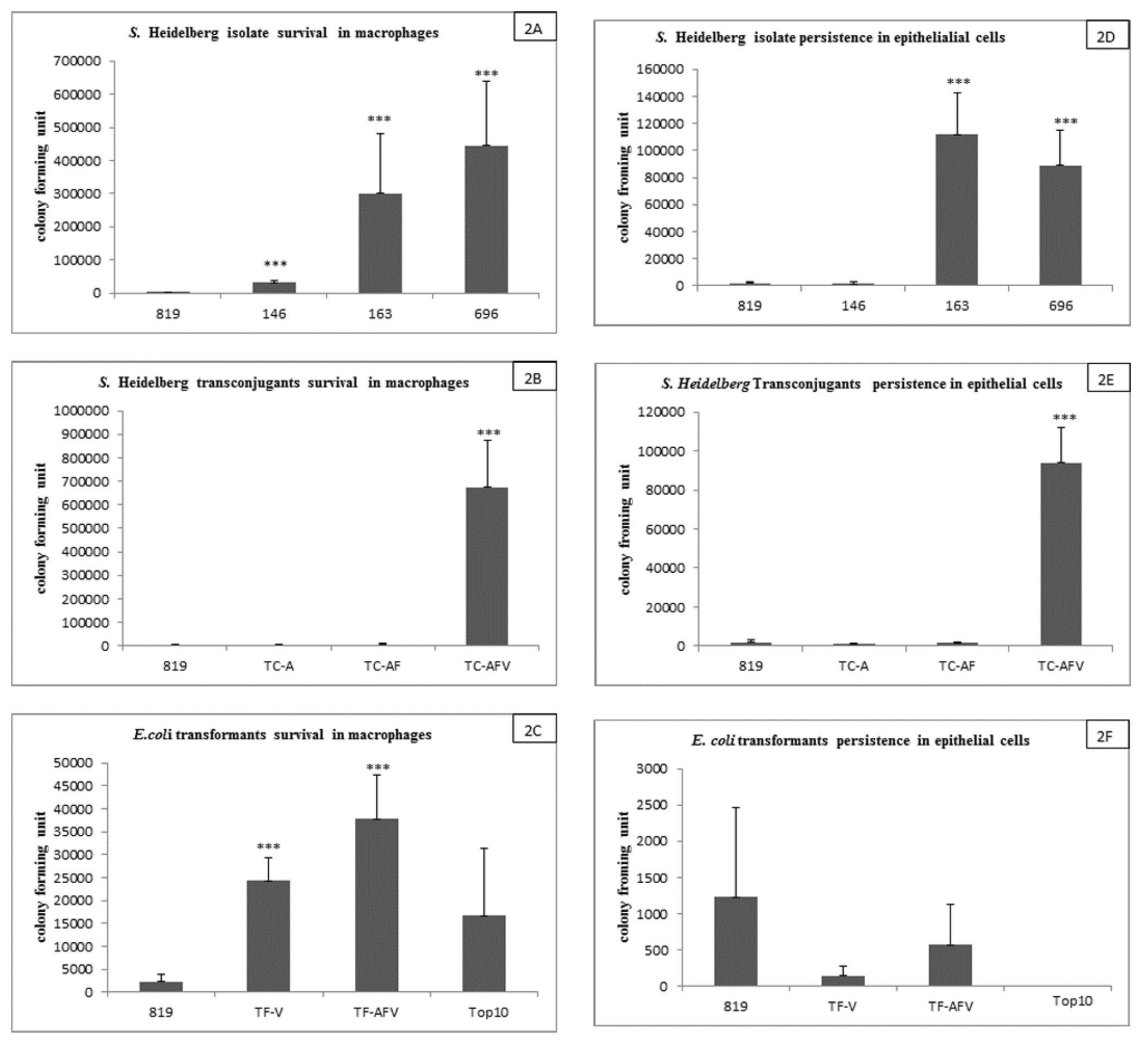
Bacterial survival assay to assess the bacterial survival mouse macrophages and epithelial cells were infected with *S.* Heidelberg strains (146, 163, 696), transconjugates and transformants that harbor transmissible plasmids and T4SS and strain that lacks both (819 and *E. coli* One Shot TOP10). Bacterial survival assays were performed 24 hr post infection. Panels A-C shows the survival in CFUs of *S.* Heidelberg isolates, transconjugants and transformants upon infection of macrophages. While panels D-F shows the number of CFUs during epithelial cell infection. Each data point represents the means and standard deviation for 5 individual experiments.

For the survival studies in the macrophages and intestinal epithelial cells, *S.* Heidelberg isolates 146, 163, 696 and TC-AFV had significantly higher numbers CFUs (each with p<0.001, respectively) than isolate 819 in the macrophage after 24 hours (Figure 2A and B). Similar results were detected in the 24-hour survival assay in the intestinal epithelial cells, where strains 146, 163, 696 and TC-AFV were significantly more able to survive than isolate 819 (each with p<0.001) (Figure 2D and E). TF-V and TF-AFV were significantly more able to survive in macrophages than 819 and the *E. coli* recipient (Figure 2C), but were less able to survive than 819 in the epithelial cells (Figure 2F).

**Figure 3:**
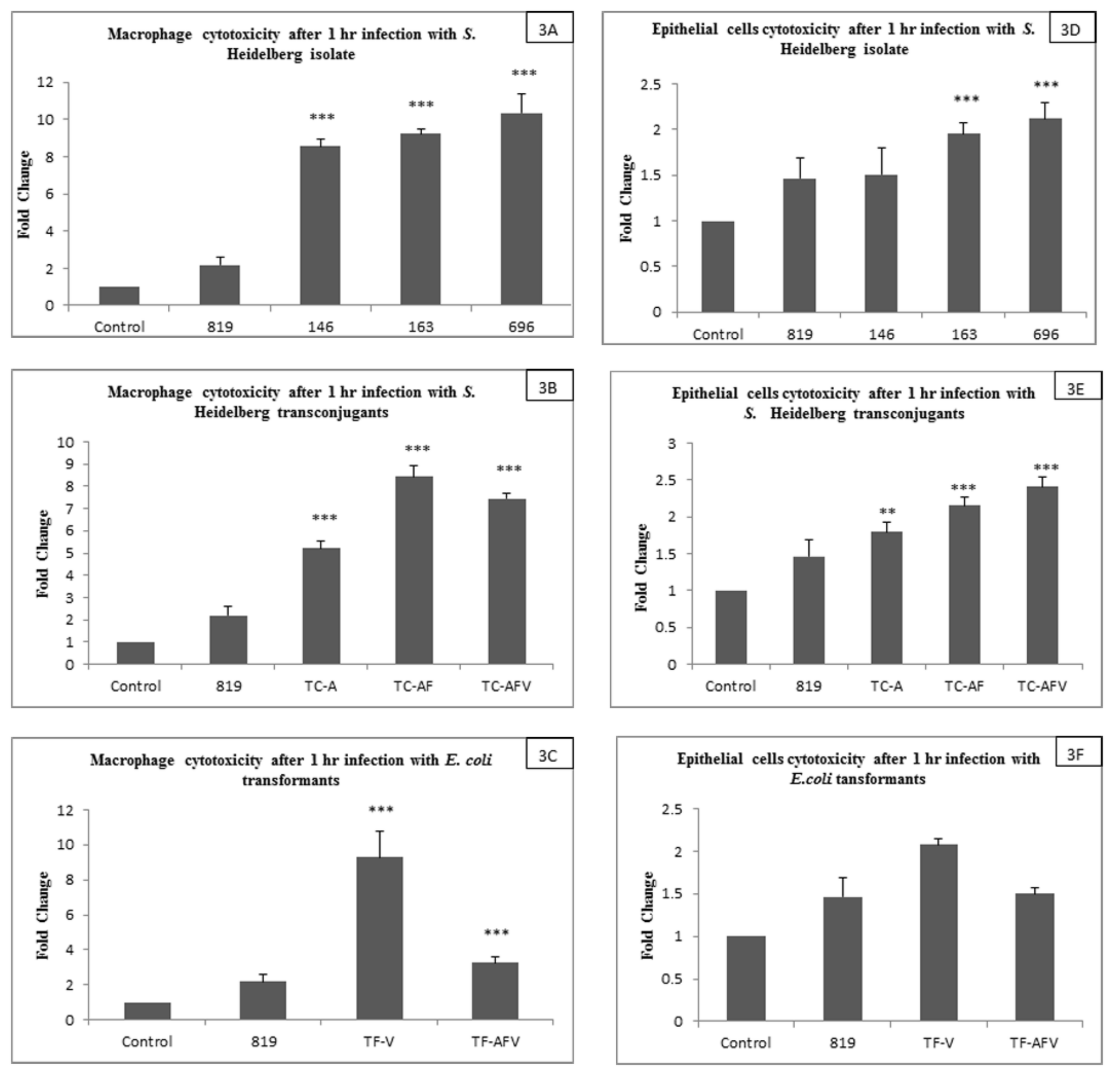
*In vitro* model to assess cytotoxocity in macrophages and epithelial cells following 1 hr infection with *S.* Heidelberg strains (146, 163, 696), transconjugates and transformants that harbor transmissible plasmids and T4SS and strain that lacks both (819 and *E. coli* One Shot TOP10). Panels A-C shows the cytotoxic effect (as determined by cytosolic LDH levels) in macrophages infected with *S.* Heidelberg isolates, transconjugants and transformants, while panels D-F shows the cytotoxic effects in epithelial cells infected with these strains. The results are expressed in fold changes as compared control among different isolates with and without mobile plasmids. Each data point represents the means and standard deviation for 5 individual experiments.

The cytotoxic effects of the *S.* Heidelberg strains and the *E. coli* transformants were determined for both macrophages and epithelial cells by assessing LDH activity. The result shows that *S.* Heidelberg isolates that harbor transmissible plasmids were able to lyse the macrophages and epithelial cells effectively, leading to a higher level of LDH activity (Figure 3). The fold increase of LDH activity was statistically significant for 146, 163 and 696 (p<0.05, p<0.001 and p<0.001, respectively) as compared *S.* Heidelberg isolate 819 (Figure 3A). The cytotoxicity results indicate that TC-AF and TC-AFV were significantly more able to kill (p< 0.01, and p<0.001, respectively) macrophages and epithelial cells compared to the recipient isolate 819 (Figure 3B and E). When the cell lysis was compared between *E. coli* transformants, the *E. coli* recipient and isolate 819; strain TF-V was significantly more able to lyse macrophages and epithelial cells (Figures C and F). However, strain TF-AFV had little effect on cell invasion and lysis in epithelial cells as compared to isolate 819 (Figure 3F).

**Figure 4:**
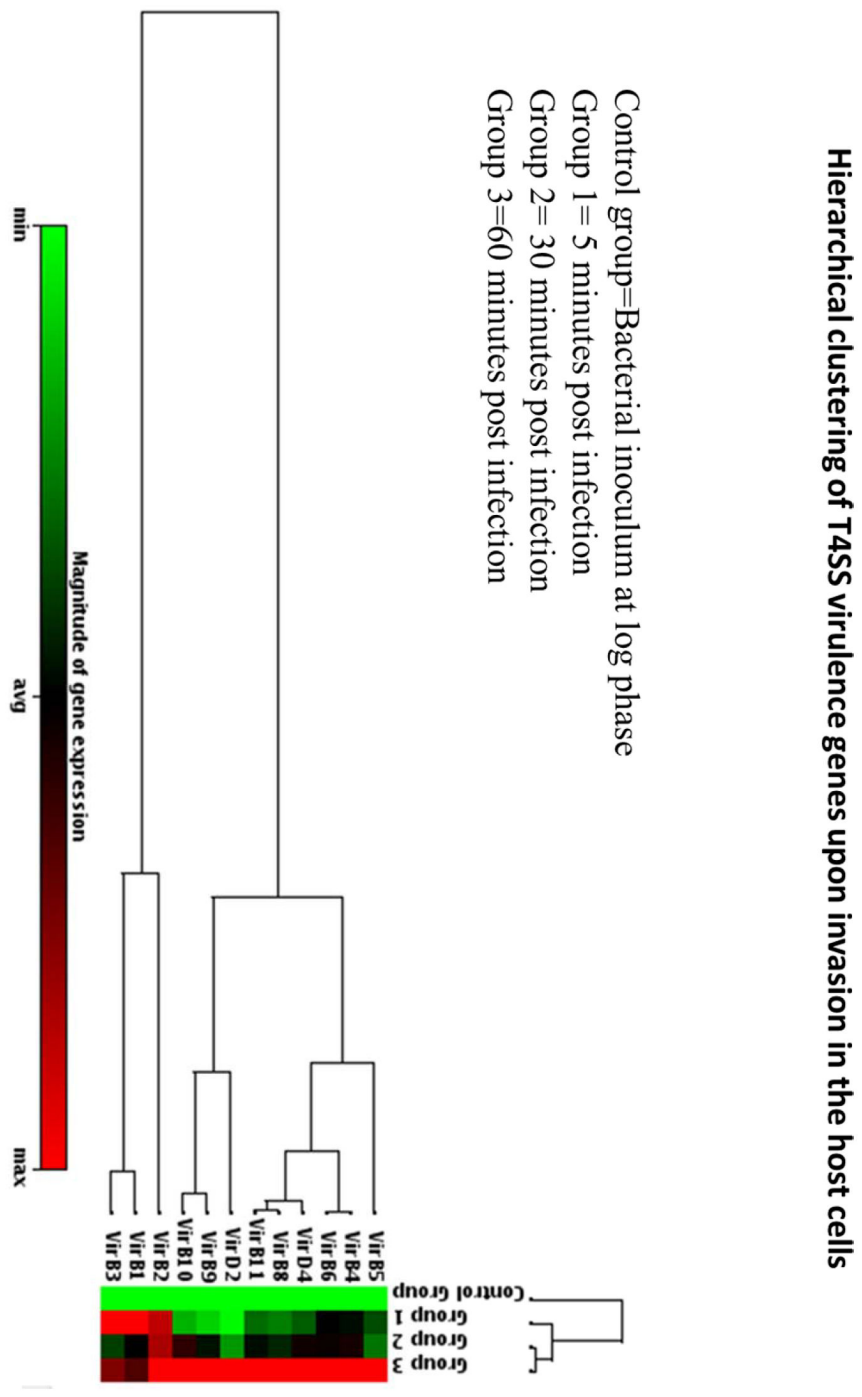
Comparative gene expression analyses of T4SS encoding genes in intracellular bacteria during macrophage infection. *S*. Heidelberg isolates 696 and 819 infected macrophage cultures for up to 1 hr. The heat map shows the gene expression profile differences at 3 different time points following infection relative to basal level (non-macrophage exposed) expression. Each data point represents the means and standard deviation for 3 individual experiments.

When the expression of T4SS genes in isolates 819 and 696 were assessed during uptake in macrophages at three different time points (5, 30 and 60 minutes post-infection) and compared to cells not inoculated with macrophages, there were altered in gene expression profiles. There was no product formed for isolate 819 throughout the experiment, since the strain lacks the VirB/D4 T4SS. For 696, at 5 minutes there was an increased level of expression of the early genes (*virB1,* 2 and 3 [sequence associated with the VirB3 domain of the annotated VirB4]) compared to original bacterial inoculum that used for infection (Figure 4). At 30 and 60 minutes there was a statistically significant (p<0.01) increase in the expression of the genes in the *virB* operon compared to the basal level expression. Data was normalized across the experiments using the 16S rRNA housekeeping gene.

**Figure 5:**
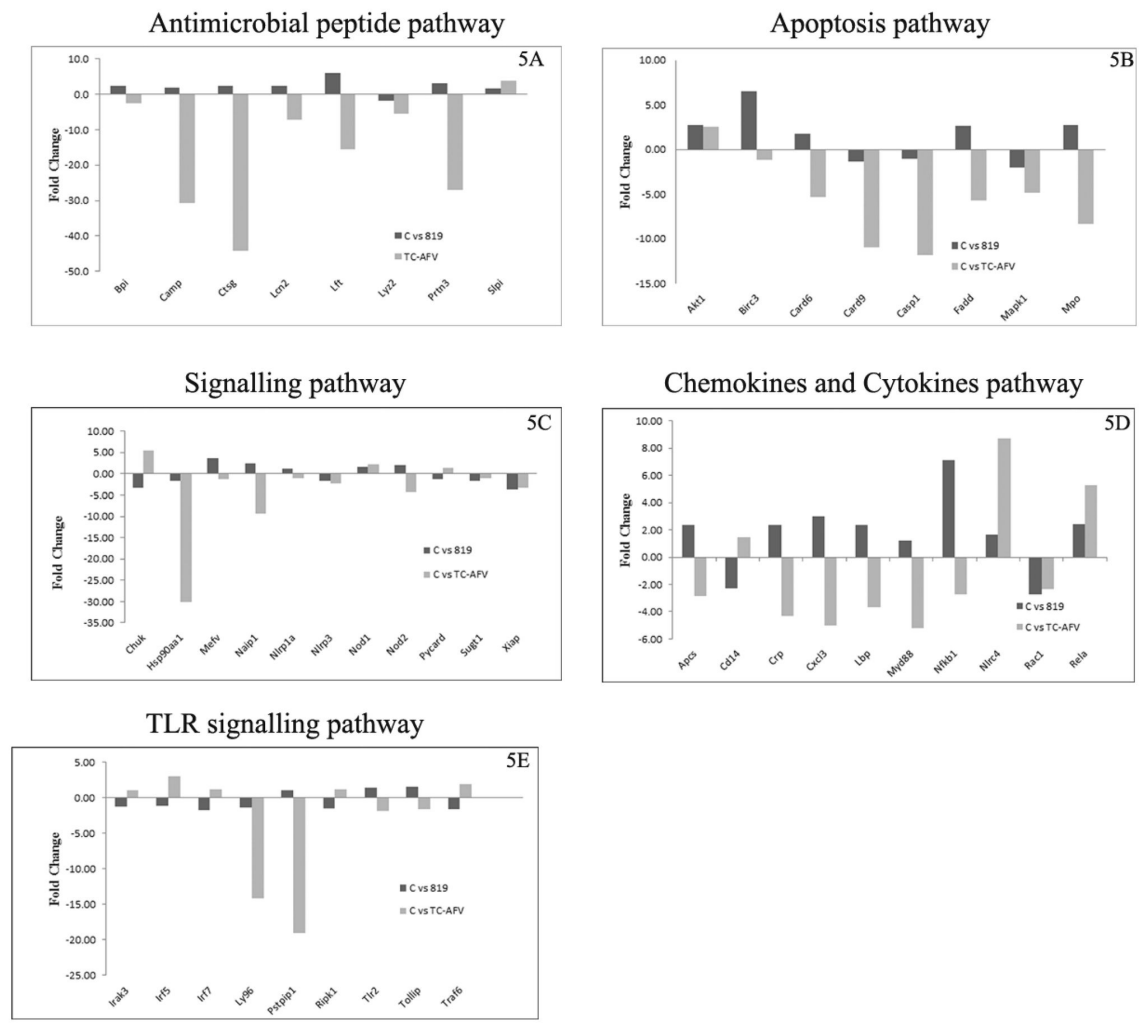
Comparison of host gene expression profile using Antibacterial Response PCR Arrays following infection with either *S.* Heidelberg transconjugant TC-AFV, which harbors mobile plasmids or the recipient isolate 819, which lack the plasmids and T4SS. Mouse macrophages were infected with TC-AFV or 819 at an MOI 1:200, for 1 hr, after which extracellular bacteria were removed and total RNA was extracted from macrophages and converted into cDNA. The pathway arrays were assessed by RT-PCR and analyzed using PCR Array Data Analysis Software (SA Biosciences). Each data point represents the means and standard deviation for 3 individual experiments.

In addition to bacterial gene expression, changes in the expression of macrophage genes associates with the antimicrobial response were evaluated using the Antibacterial Response PCR Array following the infection of macrophages with either isolate 819 or its transconjugant TC-AFV. There was a down regulation of several of the antimicrobial peptide (AMP) pathway genes by TC-AFV compared to isolate 819. The down-regulated genes were those encoding bacterial/permeability increasing protein (BPI), cationic AMP (CAMP), Ctsg, lipocalin 2 (LCn2), lactotransferin (LTF), LYZz and Prtn3 and statistically significant (p< 0.01, p< 0.001, p< 0.001, p< 0.01, p< 0.01, and p<0.01, respectively) over isolate 819 (Figure 5). There were additional alterations in the expression of some of the genes in other pathways as well including the signaling pathway, cytokines and chemokines and apoptosis pathway (Figure 5).

## Discussion

The present study was conducted to examine the role of plasmids on the invasiveness and survival of antibiotic resistant *S.* Heidelberg that were isolated from food animal sources. Each of the strains examined contained a plasmid encoding a VirB/D4 T4SS system, which has not been previously characterized in *Salmonella* to the best of our knowledge. The VirB/D4 genes were present in recently released sequences from multidrug resistant *S.* Heidelberg isolates that were associated with the 2011 ground turkey outbreak [4,33]. The VirB/D4 plasmid sequences from the isolates in the current study have been deposited in GenBank with the accession numbers JX258654-JX258656 [21]. Genes encoding VirB1-VirB11 are present with the exception of *virB7*. There is no distinct *virB3* defined in the annotation, however the *virB4* gene was predicted to encode a VirB3 domain along with VirB4. A distinct primer for the VirB3 encoding region was designed for the gene expression experiments (Table 2). In related T4SS, the *virB* genes encode for the structural and energetic subunits that assemble together and form a functional apparatus [34]. In addition, the plasmids also contain genes for VirD2 and VirD4, which are likely involved in recruiting secreted effector molecules and activating the T4SS, respectively [34]

To determine the relative impact of different plasmids on virulence, wild type Salmonella isolates and a series of transconjugants and transformants were utilized. The plasmids in these strains were previously sequenced [21] and each of the wildtype isolates examined contained two plasmids encoding antimicrobial resistance determinants (IncA/C, IncFIB and/or an untypeable plasmids) as well as a plasmid that appeared to encode a VirB/D4 T4SS. The IncA/C plasmids have been found in several *Salmonella* serotypes and are known to be important for multidrug resistance [35,36] and the IncFIB plasmids have been identified in *Salmonella* serotypes common to poultry and in avian pathogenic *E. coli* [21,28]. These IncFIB plasmids often contain likely virulence genes, in addition to the antimicrobial resistance determinants [28].

Overall, the results of the study indicate that the VirB/D4 T4SS-containing plasmid encodes for an enhanced ability of *S.* Heidelberg to invade and persist in macrophages and intestinal epithelial cells. T3SSs likely also contributed to the virulence, which was evident with isolate 819 that was able to enter and survive in the cell lines, but at a lower level than the VirB/D4 T4SS containing strains. Each of the isolates demonstrated a similar level of expression of SPI 1 and 2 T3SS-associated genes, which supports that notion that the majority bacterial uptake of strain 819 by macrophage/epithelial cells was mediated by T3SSs. In contrast, a relatively small portion of bacterial uptake of strains 146, 163, 696 and transconjugates was likely due to T3SSs; with much of the rest due to plasmid-associated factors, such as the VirB/D4 T4SS. Indeed the *E. coli* transformants, which lack SPI 1 and 2, yet contained the VirB/D4 plasmid, were able to be taken up and survived in macrophages to a significantly greater extent than isolate 819.

The results of the bacterial survival experiment implicate the involvement of both IncA/C and IncFIB plasmids in bacterial survival in macrophages. In the absence of the VirB4/D4 plasmids, *S.* Heidelberg isolates were able to invade macrophages; however, they were unable to survive for 24 hours in macrophages. The other plasmids, such as the IncI1, untypeable resistance and small (<5 kb) crytic plasmids, in the strains appeared to play a limited role in bacterial uptake of macrophages and epithelial cells. The IncA/C plasmids encode a number of known antimicrobial resistance genes, but have a large number of coding sequences for hypothetical proteins that may potentially contribute to virulence in some aspect. The IncFIB plasmids have been associated with bacterial virulence in avian hosts, most notably for their roles in extraintestinal survival due in part to the iron acquisition operons and in some cases serum and complement resistance [28,37]. In the *E. coli* transformants, TF-V demonstrated a significantly greater ability to invade the macrophages than TF-AFV, this is potentially the results of a fitness cost associated with the carriage of two additional large plasmids (~120 and 135 kb) in their non-native or adapted host. The role of fitness associated with the carriage of plasmids was not directly evaluated in the current study, but may be an important issue to address in future studies on the impact of plasmids on bacterial virulence.

The cytotoxicity experiments indicate that the strains containing the VirB/D4 plasmids had an increased ability to lyse the host cells during initial infections; however, in the cells that survived, the *Salmonella* were able to enter and survive at higher rates that those without the T4SS containing plasmids. Indeed T4SSs have been identified for their importance in several pathogenic bacterial species to survive in hostile environments such as within macrophages [25,38]. The *S*. Heidelberg VirB/D4 plasmid sequences contained each of T4SS genes, with the exception of *virB7*, that have been reported in orthologous T4SS in other bacterial species [39,40]. Intracellular pathogenic bacteria employ T4SS gaining entry and persist within the host [27,41,42] and potentially serve as a reservoir for zoonotic transmission [38]. The impact of T4SS on *Salmonella* survival in host is potentially associated with the SCV. Earlier studies have shown that the expression of T4SS encoding genes is up-regulated in intracellular *Brucella* and *Burkholderia cepacia* during infection with macrophages and result in the bacteria bypassing normal lysosomal fusion and subsequent killing by macrophages [43,44]. The T4SS genes are expressed within bacterial containing vacuoles (BCV) which interact with the ER to avoid the endocytic killing mechanism initiated by macrophages, thereby suppressing the innate immune response [26]. The survival of *S.* Heidelberg isolates 146, 163, 696 and transconjugates in macrophages, coupled with the observed up-regulation of the *virB/D4* genes in isolate 696 during infection in cultured macrophages, correlate with the earlier findings of the contribution of the T4SS for survival within macrophages [26,43]. These findings highlight the potential for the VirB/D4 T4SS to serve as a crucial pathogenic factor for *S.* Heidelberg to increase its ability to cause infections in livestock and potentially humans.

The T4SS secretary machinery is a multicomponent protein complex and can be grouped into three units based on their assembly and functional properties, which include 1) pilus-associated components consists of VirB2, VirB3 and possibly VirB5, 2) channel forming core unit that include VirB6-VirB10 and 3) coupling components consisting of ATPases (VirD4, VirB4 and VirB11). The gene expression analysis of present study show that they grouped into these three basic components. At the early stage infection (after 5 minutes infection), the pilus-associated genes are highly expressed relative to at 30 and 60 minutes post infection. Conversely, at 30 minutes post infection, the majority of core units and coupling units are more highly expressed than at 5 minutes (Figure 4). At 60 minutes post infection the expression of core components and coupling units are significantly greater that the earlier two time points. The overall timing of the gene expression differences during infection process likely reflects the progression of the formation of functional T4SS in *S.* Heidelberg. For example, the early expression of *virB2* gene, which encodes a key component of the core pilus structure [34], likely reflects the importance of this protein in the initial assembly of the T4SS.

Bacterial secretion systems (both T3SS and T4SS) are known to translocate a variety of difference virulence factors, effector proteins, flagellin, peptidoglycan, nucleic acids into the host cells that can activate the innate immune response signaling pathways to produce cytokines, and proinflammatory molecules [45-47]. *In vivo* studies have shown that *B. abortus* and *B. melitensis* T4SSs are essential to activate the expression of genes associated with cytokines and chemokines [48]. The current study utilized the Antibacterial Response PCR Array for mouse macrophages to better understand how the VirB/D4 T4SS-containing *S.* Heidelberg isolate interacts with host cells at early stage of bacterial uptake, and how it modifies transcriptional profile of genes in macrophages during bacterial uptake and colonization. Genes analyzed with the Antibacterial Response PCR Array were categorized into six groups, including AMPs, apoptosis associated factors, cytokines and chemokines, inflammatory response, signaling molecules and toll like receptor (TLR) signaling pathways. The goal of using the pathway array was to identify the pathways are highly altered during early stage of bacterial uptake. Strain TC-AFV and the recipient 819 were chosen for the experiments because the use of the transconjugant would allow for a more direct measure of the impact of the plasmids on the host cell responses. One hour post infection, TLR signaling, apoptosis and chemokine and cytokine signaling pathway genes were generally down-regulated in macrophages infected with TC-AFV. Our finding is similar to the earlier findings, in which T4SS alters signal transduction in host cells during infection process [49]. The pathway that was maximally impacted was the AMP pathway and signaling pathways which play vital roles in the innate immune system.

TC-AFV altered the expression of several genes that are involved with the initial interaction between host and pathogen. Genes encoding BPI, CAMP, Ctsg, LCn2, LTF, LYZz, and Prtn3 were down-regulated in TC-AFV compared to the recipient 819. These genes are likely important for the host to mount the innate immune response in macrophages. The crucial function of BPI is binding with LPS on the surface of bacteria through the charged residues that are located on the surface of the protein [50]. BPI is able to insert into the bacterial cell wall due to its hydrophobic core, increasing the cell wall permeability and leading to hydrolysis by the bacterial cell wall enzymes [51]. LTF is a non-heme iron binding protein and an essential component of the innate immune system [52]. It executes various protective functions for the host destabilizing bacterial membrane, inhibiting bacterial multiplication by altering membrane structure, enhancing the immunostimulatory effects, and binding with bacterial lipid A molecules [53]. Earlier studies have also shown that LTF enhances the antigen specific IgG, IgA and IgM secretion [54]. In addition, LTF has also been shown to help protect animals challenged with *Salmonella* and to decrease bacterial load in tissues [55] and to decrease the bacterial adherence in HeLa cells [56]. Bovine LTF exhibits antimicrobial effects upon incubation with *S. Enteritidis* and *P. fluorscens* [57].

The protein LCn2 is an antimicrobial protein and exhibits bacteriostatic properties. The major source of LCn2 is intestinal epithelial cells, macrophages and other immune cells. *In vivo* and *in vitro* studies have demonstrated that LCn2 arrest the bacterial growth by limiting the availability of iron, which is an essential trace element for bacterial growth and cellular function [58-60]. A recent *in vivo* study showed that *S. Typhimurium* infection stimulates the secretion LCn2 in the inflamed lumen of rhesus monkeys [61]. An earlier study had shown that the MyD88 dependent signaling pathway up-regulates LCn2 induction [62]. The gene expression data shows that *S.* Heidelberg TC-AFV down regulates MyD88 gene expression (Figure 5), which is consistent with the LCn2 expression results, however further studies are needed to correlate the altered expression of LCn2 with decreased level of the MyD88 signaling pathway.

Overall, the expression array results indicate that the AMP pathway was altered in macrophages after infection with the *S.* Heidelberg isolate containing the plasmids (TC-AFV). AMP molecules are the first line defense system of innate immunity. AMP molecule secretion is generally initiated in two ways: 1) microbial derived molecules such as LPS activate innate immune receptors, and 2) activated immune cells mediate signals for the secretion of AMP peptides. The down-regulated AMP pathway genes could lead to decreased expression of AMP peptides. The final outcome of this event is uptake of bacteria in the host immune cells. Our study provides the evidence for a role of plasmid encoded genes in the alteration of gene expression profiles that facilitates invasion of macrophages.

## Conclusions

This study provides several important findings related to the pathogenicity of *S.* Heidelberg and the role of plasmids in virulence. *S.* Heidelberg strains can contain multiple plasmids that are important for antimicrobial resistance and virulence. Each of the strains examined contained a plasmid encoding a VirB/D4 T4SS, which had not been previously evaluated in *Salmonella*. Based on the results of bacterial uptake and survival, the T4SS-containing plasmid enhances the ability of *S.* Heidelberg strains to invade and survive in intestinal epithelial and macrophage cell lines. The expression of the secretion system genes during entry into macrophages and the impact of plasmid acquisition on survival indicate that the T4SS is likely an important attribute to enhance the virulence of the organisms. The role in virulence is likely due to the modulation of the host immune response, since the plasmid-containing strains down-regulated multiple members of the AMP pathways, which have been associated with limiting killing by the immune system components. The overall results of the study demonstrate the potential importance of the VirB/D4-T4SS containing plasmids in virulence of *S.* Heidelberg and potentially other organisms as well.
